# Porous insights: IpaH7.8 reveals crystal-clear differences of gasdermins in mice and men

**DOI:** 10.1038/s41392-023-01535-z

**Published:** 2023-06-28

**Authors:** Friedrich Alexander von Samson-Himmelstjerna, Benedikt Kolbrink, Stefan Krautwald

**Affiliations:** Department of Nephrology and Hypertension, University Hospital Schleswig-Holstein, 24105 Kiel, Germany

**Keywords:** Inflammation, Translational research

Two recent back-to-back papers published in *Nature* provide new insights into the structural basis of gasdermin B (GSDMB) targeting by the secreted *Shigella* virulence effector IpaH7.8, the regulation of GSDMB’s pore-forming pyroptotic activity by alternative splicing, and into the inter-species differences regarding IpaH7.8-mediated inhibition of gasdermin D (GSDMD) activity.^1,2^

Pyroptosis is a mode of inflammatory regulated cell death (RCD) that plays a critical part in the innate immune response to invasive pathogens. It is executed by a family of structurally related pore-forming proteins known as gasdermins, of which GSDMD is best studied as the canonical executor of pyroptotic cell death in macrophages upon activation of the NLRP3 inflammasome and subsequent cleavage of caspase-1.^3^ GSDMB is currently one of the lesser studied members of this family, and little is known about its functional relevance and pyroptotic potential during active disease states. It is speculated that in certain circumstances, pathogens aim at inhibiting the host’s RCD mechanisms to sustain the pathogen’s environment for replication. Interestingly, the ubiquitin-ligase IpaH7.8 has been reported to degrade GSDMs and thereby serve as a virulence factor secreted by *Shigella*, a pathogenic Gram-negative bacterium, but there has been inconsistent evidence on whether GSDMB and GSDMD are both targeted equally, and whether IpaH7.8 interaction diverges between the two GSDMs.

To resolve this question, both Wang et al.^1^ and Zhong et al.^2^ performed cryo-electron microscopy (EM) of the IpaH7.8–GSDMB complex. They identified a leucine-rich repeat motif in IpaH7.8 responsible for the key interaction between IpaH7.8 and the *N*-terminal domain of GSDMB. Subsequently, conservation of this motif was observed by both groups in human GSDMD, but not in mouse GSDMD. As opposed to human GSDMD, neither Wang et al. nor Zhong et al. could detect an interaction between IpaH7.8 and murine GSDMD in their co-localization studies. Thus, both groups were able to precisely demonstrate the mechanism of S*higella*-induced inhibition of pyroptosis.

Recently, several other pathogen-induced resistance mechanisms to host-induced RCD have been reported. NleB1 is a notable example reminiscent of the interaction between IpaH7.8 and GSDMs: This secretion system effector with *N*-acetylglucosamine transferase activity can block another important pathway of RCD, necroptosis, which shares many common pathway components with pyroptosis.^4^ NleB1 is secreted by enterohaemorrhagic *Escherichia coli* and binds to host Fas-Associating Death Domain-Containing Protein, subsequently antagonizing death receptor signaling by preventing caspase-8 activation. Studies such as those of Wang et al. and Zhong *et al*. provide potential cues for the future development of novel antimicrobial strategies; in light of the increasing spectrum of multidrug resistant bacteria, this offers a glimmer of hope that should encourage further investigation.

Another fascinating—or perhaps disturbing—matter is implicitly addressed by the comparison between human and mouse GSDMD performed in these studies: They make us painfully aware of the fine but apparently critical differences between key players of RCD pathways in mice and humans, and that these variances completely change the response to signal induction and transduction. Throughout the past centuries many of these interspecies alterations were uncovered and they include a wide variety of mechanisms. Why is this disturbing? Because vast amounts of preclinical evidence on inhibitors of RCD have been accumulated, largely derived from studies with rodents. The utter failure of the billion-dollar development of RIPK1 and RIPK3 inhibitors for human treatment makes clear that we have not identified the necessities to translate our knowledge from rodents to humans. More optimistically, works such as those of Wang et al. and Zhong et al. will hopefully help us close the gap, and enable future success of the bench-to-bedside, animal-to-human approach in this field.

**Fig. 1:**
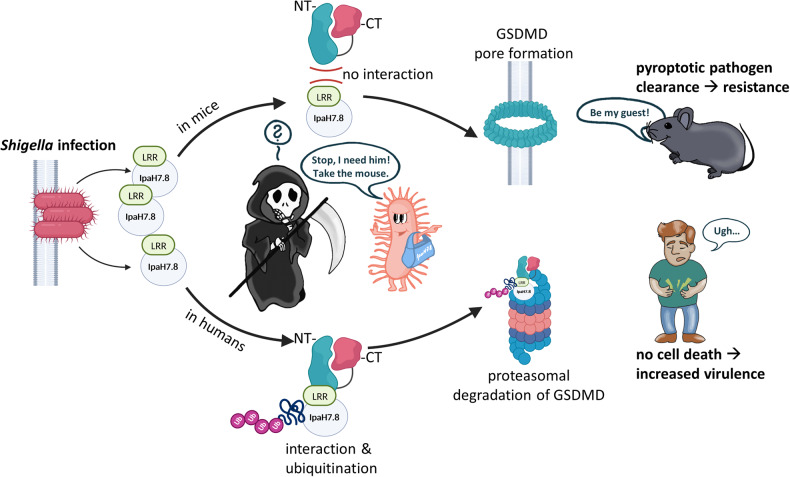
The devil is in the details. After unveiling that the *Shigella* virulence effector IpaH7.8 (with purse) inhibits the pore-forming pyroptotic activity of GSDMB by interacting with a leucine-rich repeat (LRR) motif at the *N*-terminal domain of GSDMD, it was discovered that this motif is conserved in humans but not in mice.^1,2^ This permits *Shigella* to evade innate pathogen clearance (grim reaper) in humans and to maximize its reproduction in enteric cells, increasing its virulence (man with abdominal pain). Meanwhile, mice are resistant to shigellosis, using immunogenic regulated cell death to their benefit (carefree mouse). BioRender is used to generate part of the figure

The investigators characterized different GSDMB isoforms separately and solved the pore structure of GSDMB by using single-particle cryo-EM. The structure of the GSDMB-IpaH7.8 complex reveals the presence of an acidic motif composed of three negatively charged residues (E15, D17, and D21) located in the *N*-terminal α1-β1' loop of both GSDMB and human GSDMD. This acidic motif serves as the key structural element specifically recognized by *Shigella* IpaH7.8. However, it is important to note that IpaH7.8 does not bind to murine GSDMD, as the α1-β1' motif is not conserved in mice. As a result, *Shigella* is unable to efficiently establish infection in mice (Fig. 1). Furthermore, they demonstrated that alternative splicing regulates the interdomain linker configuration as well as pore formation ability. The exon 6-encoded sequences in the *N*-terminal domain of GSDMB dictates its pore-forming, pyroptotic activity, highlighting that different isoforms have varying capability to induce pyroptotic membrane rupture.

Interesting new research ideas beyond bacteriology could develop from these findings on the different properties of isoforms of GSDMB. Oncology appears to be a particularly interesting field here, as immunogenic cell death—such as pyroptosis—has recently received significant attention in the context of cancer.^5^ The relevance of RCD in this field is also illustrated, for example, by the fact that the screening of cancer cell lines played a critical role in the identification of glutathione peroxidase 4 as the key regulator of ferroptosis, a mode of RCD characterized by oxidative stress and lipid peroxidation.^3^ As Zhong et al. report, different cancer cell lines express different isoform compositions of GSDMs. The greatly varying activity of the different GSDMB isoforms in this work suggests that epigenetic and post-transcriptional factors need to be considered urgently in the design of studies regarding RCD targets for tumor treatment. Moreover, the study of Zhong et al. gives us a hint as to how this finding of differentially active isoforms of GSDMB could be used therapeutically: A number of cytokines regulate the expression of GSDMB isoforms relevant to pyroptosis. Based on this knowledge, it seems reasonable to investigate new therapeutic approaches that may increase the vulnerability of malignant cells to highly immunogenic cell death.

Overall, both studies published in *Nature* illustrate the fine regulation of GSDMB pore formation by infectious bacteria and decipher alternative mRNA splicing of the interdomain linker in GSDMB as a molecular determinant and regulator of GSDMB isoforms-dependent pyroptotic activity. These structural insights are novel and very interesting in their own right, and beyond that have potentially important implications for translational approaches.
